# Reduced *Brd1* expression leads to reversible depression-like behaviors and gene-expression changes in female mice

**DOI:** 10.1038/s41398-020-00914-2

**Published:** 2020-07-17

**Authors:** Anto P. Rajkumar, Per Qvist, Julie G. Donskov, Ross Lazarus, Jonatan Pallesen, Nicoletta Nava, Gudrun Winther, Nico Liebenberg, Sanne H. la Cour, Veerle Paternoster, Tue Fryland, Johan Palmfeldt, Kim Fejgin, Arne Mørk, Mette Nyegaard, Bente Pakkenberg, Michael Didriksen, Jens R. Nyengaard, Gregers Wegener, Ole Mors, Jane H. Christensen, Anders D. Børglum

**Affiliations:** 1 IPSYCH, The Lundbeck Foundation Initiative for Integrative Psychiatric Research, Aarhus, Denmark; 2 Department of Biomedicine and Centre for Integrative Sequencing, iSEQ, Aarhus University, Aarhus, Denmark; 3 Center for Genomics and Personalized Medicine, Aarhus University, Aarhus, Denmark; 4 Division of Psychiatry and Applied Psychology, University of Nottingham, Nottingham, UK; 5 Department of Old Age Psychiatry, Institute of Psychiatry, Psychology, & Neuroscience, King’s College London, London, UK; 6 Synaptic Transmission, H. Lundbeck A/S, Copenhagen, Denmark; 7 Computational Biology, Baker IDI Heart and Diabetes institute, Melbourne, VIC Australia; 8 Translational Neuropsychiatry Unit, Department of Clinical Medicine, Aarhus University Hospital, Aarhus, Denmark; 9 Core Centre for Molecular Morphology, Section for Stereology and Microscopy, Department of Clinical Medicine, Centre for Stochastic Geometry and Advanced Bioimaging, Department of Clinical Medicine, Aarhus University, Aarhus, Denmark; 10 Research Unit for Molecular Medicine, Aarhus University Hospital, Aarhus, Denmark; 11 Research Laboratory for Stereology and Neuroscience, Bispebjerg University Hospital, Copenhagen, Denmark; 12 Psychosis Research Unit, Department of Clinical Medicine, Aarhus University Hospital, Aarhus, Denmark

**Keywords:** Epigenetics and behaviour, Depression, Molecular neuroscience

## Abstract

The schizophrenia-associated gene, *BRD1*, encodes an epigenetic regulator in which chromatin interactome is enriched with genes implicated in mental health. Alterations in histone modifications and epigenetic regulation contribute to brain transcriptomic changes in affective disorders and preclinical data supports a role for BRD1 in psychopathology. However, the implication of BRD1 on affective pathology remains poorly understood. In this study, we assess affective behaviors and associated neurobiology in *Brd1*^+/−^ mice along with their responses to Fluoxetine and Imipramine. This involves behavioral, neurostructural, and neurochemical characterizations along with regional cerebral gene expression profiling combined with integrative functional genomic analyses. We report behavioral changes in female *Brd1*^+/−^ mice with translational value to depressive symptomatology that can be alleviated by the administration of antidepressant medications. Behavioral changes are accompanied by altered brain morphometry and imbalances in monoaminergic systems. In accordance, gene expression changes across brain tissues reveal altered neurotransmitter signaling and cluster in functional pathways associated with depression including ‘Adrenergic-, GPCR-, cAMP-, and CREB/CREM-signaling’. Integrative gene expression analysis specifically links changes in amygdaloid intracellular signaling activity to the behavioral treatment response in *Brd1*^+/−^ mice. Collectively, our study highlights the importance of BRD1 as a modulator of affective pathology and adds to our understanding of the molecular mechanisms underlying affective disorders and their treatment response.

## Introduction

Psychiatric disorders comprise a heterogeneous group of disabling conditions collectively characterized by self-reported and clinically observed changes in state of well-being and abnormal behaviors^1^. Suggestive of interconnected etiologies, clinical, and therapeutic profiles are overlapping, and risk factors are shared between disorders^2,3^. In line with the neurodevelopmental hypothesis of psychiatric disorders^4^, this includes a complex interplay between genetic risks^5^ and early life adverse exposures^6^. However, the molecular and biological mechanisms that trigger early life programming and development of psychopathology are poorly understood. Epigenetic processes, such as acetylation of histone lysine residues, are linked with brain development as well as lifelong neural plasticity^7^ and have been implicated with the pathophysiology of both psychotic and affective disorders^8^. Correspondingly, a number of clinically effective antidepressants are known to affect the status of cerebral acetylation of histone lysine residues (Kac)^9–11^ and histone deacetylases (HDACs) have been suggested as direct therapeutic targets for depressive disorders^12,13^. The interpretation of Kac marks is facilitated by the reader domains of the bromodomain (BRD) family of proteins^14^. Whereas Kac is generally associated with activation of transcription through opening of the chromatin structure, they may also signal for the compaction of chromatin, protein stability, and the regulation of protein-protein interactions^15^.

Bromodomain containing-1 (BRD1) has been identified in complexes involved with both histone acetylation and chromatin remodeling^16,17^ and interacts at genomic sites enriched with genes implicated in neurodevelopmental processes^17,18^. *BRD1* is widely expressed in human brain^19^, differentially regulated in limbic and neocortical tissues upon exposure to external stressors in rats^20,21^, and involved in the epigenetic regulation of embryonic development, survival, and differentiation of embryonic stem cells^16,22,23^. Supporting a role for *BRD1* in mental health, *BRD1* has repeatedly been associated with schizophrenia and bipolar disorder in genetic studies^24–28^, including gene-wise significant association in the currently largest schizophrenia GWAS mega-analysis^29^ and genome-wide significance in the Psychiatric Genomics Consortium (PGC1) schizophrenia sample^30^. The locus does not show significant association in the most recent GWASs of bipolar disorder and major depressive disorder^31,32^. Despite being highly intolerant to loss of function mutations^33^, a disruptive nonsense mutation in *BRD1* has been reported in a schizophrenia case^34^. In concordance, we have recently shown that male mice with reduced expression of *Brd1* (*Brd1*^+/−^ mice) recapitulate cardinal features relating to schizophrenia^35–38^ (Table S1). In the present study, we assess the impact of reduced *Brd1* expression on affective symptomatology, neurochemistry, and neurobiology in mice along with their responsiveness to pharmacological intervention using clinically effective antidepressants. Through global gene expression profiling of selected brain regions, we assess the neuromolecular mechanisms that underly BRD1’s role in affective pathology.

## Materials and methods

### Animals

A mouse line heterozygous for a targeted deletion within the *Brd1* gene, C57BL/6NTac-Brd1tm1.2Arte/AborgMmucd (*Brd1*^+/−^) was generated by TaconicArtemis GmbH (Cologne, Germany) using a targeting vector (p*Brd1* Final cl 1 (UP0257)) with loxP sites flanking exon 3–5 of the *Brd1* gene. For further details, see Supplementary Methods and Materials. The mouse strain has been deposited and is available at Mutant Mouse Resource and Research Center (MMRRC) at University of California at Davis (RRID:MMRRC_065563-UCD). All studies were carried out in accordance with Danish legislation, and permission for the experiments was granted by the animal welfare committee, appointed by the Danish Ministry of Food, Agriculture and Fisheries—Danish Veterinary and Food Administration.

### Experimental design

All experiments involved 7–15 mice, 8–11 weeks old, in each group (figure legends present exact numbers). The sample size was chosen on the basis of the resource equation method^39^. Litter-matched WT and *Brd1*^+/−^ mice were randomly allocated to individual tests. Observer was blind to mice genotypes. General assessment of neurology, testing in motor coordination tests, prepulse inhibition (PPI), fear conditioning (FC), 8-arm radial maze (8ARM), open field (OF), tail suspension test (TST), forced swim test (FST), bright open field (BOF), light and dark box (LDB), elevated plus maze (EPM) and quantification of neurotransmitters by high-pressure liquid chromatography (HPLC) were performed in parallel on age-matched male and female littermates. However, in this study, PPI, FC, and 8ARM were only reported in female mice, since corresponding male data have already been published elsewhere^35,36^. One batch of mice completed OF, TST and FST, while another completed BOF, LDB, and EPM. An independent batch of mice was injected subcutaneously with either vehicle (normal saline solution), imipramine (IMN, 1 or 10 mg/kg) or fluoxetine (FLX, 5 mg/kg), dissolved in saline and subjected to OF, TST, and FST. Each mouse underwent 2 days of experiments. Day 1: one hour after the injection, the mice completed TST and were subsequently returned to their cages. Day 2: mice had a second injection. One hour after the injection, the mice completed OF and FST. There was no delay between OF and FST. One hour after the completion of FST, they were sacrificed, and brains were collected.

Amphetamine-induced hyperactivity (AIH), cocaine-induced hyperactivity (CIH), sucrose preference test (SPT), and 24-h locomotor activity (24HLM) and OF, TST and FST following administration of antidepressants were only tested and reported in female mice in this study. Mice were not reused for other experiments.

### General assessment of neurology, motor coordination, and behavioral tests

Details on functional observation battery, acute pain response, rotarod, balance beam walking, foot-printing, FST, TST, SPT, OF, BOF, LDB, EPM, FC, 8ARM, 24HLM, PPI, AIH, and CIH can be found in Supplementary Methods and Materials.

### Quantification of neurotransmitters

Mice were sacrificed by cervical dislocation and frontal cortical, hippocampal, and striatal tissues were collected by free-hand dissection and processed for quantitative HPLC analyses of dopamine and serotonin. For details on HPLC procedures, see Supplementary Methods and Materials.

### Brain morphometry, Golgi-cox staining, and 3-D image analysis

Left cerebral hemispheres (*n* = 8/group) were stained with FD Rapid Golgi-Stain kit (FD Neurotechnologies, Ellicott City, USA), and cut into 150 µm thick-slices on a vibratome-3000 (Vibratome, St Louis, MO, USA). Anterior cingulate cortex (aCC) pyramidal neurons were identified (×60; oil-immersion; numerical-aperture = 1.4) by their prominent apical dendrites, and 6 neurons/mouse were chosen by systematic uniform random sampling. Image stacks (90-105 consecutive images at 1 µm interval) were captured by optical wide-field microscopy (Olympus BX50, Tokyo, Japan) and newCAST software (Visiopharm, Hoersholm, Denmark). 3-D image reconstruction and analyses were completed using Imaris software version 7.6.3 (Bitplane AG, Zurich, Switzerland). For a description on brain morphometric analyses, see Supplementary Methods and Materials.

### Statistical analysis

STATA 15.1 (StataCorp LLC, College Station, TX) and GraphPad Prism 8.1.2 (GraphPad Software, San Diego, CA) software was used for analyzing our data with appropriate tests of statistical significance including *t*-test and two-way RMANOVA. We checked whether all continuous variables followed Gaussian distribution using Shapiro–Wilk tests. When the study variables did not follow Gaussian distribution, appropriate non-parametric tests such as Mann–Whitney *U* test were employed. *F* tests were used for assessing equality of variances and Welch corrections were applied, when needed.

### RNA-sequencing and data analyses

Mouse brains from IMN (10 mg/kg) and FLX (5 mg/kg) (*n* = 9–10/group) were sectioned coronally (1 mm thick) using a slicer matrix (Zivic Instruments, Pittsburgh, USA). For the IMN (10 mg/kg) group, right amygdala (AMG), striatum, caudate putamen (CPu), and aCC were identified, and punched by a punch-needle (1 mm diameter) at −20 °C, whereas for the FLX (5 mg/kg) group only AMG was sampled. RNA was extracted using Maxwell-16 instrument system and LEV simplyRNA Tissue Kit (Promega, Madison, USA). Agilent 2100 Bioanalyzer (Agilent technologies, SantaClara, USA) confirmed the quality of RNA with a mean RNA Integrity Number (RIN) of 7.87 (SD 0.26). For AMG samples, libraries were prepared using TruSeq library preparation kit and RNA-sequencing performed on the Illumina HiSeq2000 platform (Illumina, San Diego, USA). For CPU and aCC samples, libraries were prepared using Beijing Genomics Institute (BGI) library preparation kits and protocols and sequencing performed on the BGISEQ-500 platform. A minimum of 10 million clean 50 bp single-end reads were generated for each sample. Reads that passed quality control (more than 90% bases having less than 1% sequencing error; No ambiguous bases) were aligned to mouse genome (Mus_musculus.GRCm.38.90) by HISAT2 (version 2.1.0)^40^ and counted by StringTie (version 1.3.4)^41^. Differentially Expressed Genes (DEGs) were identified by edgeR3.24.1^42^ and reported after Benjamini-Hochberg false discovery rate (FDR) (5%) correction (14, 16) or as nominally significant DEGs (*p* < 0.01). Functional analyses of all nominally significant DEGs were performed using Ingenuity Pathway Analysis (Ingenuity, Redwood City, USA).

## Results

### General assessment of neurology and motor coordination

Male and female *Brd1*^+/−^ mice were overall healthy, as described elsewhere^35^. However, female mice showed marginally impaired growth and slightly reduced size^35^. Systematic testing of general neurological functions of female *Brd1*^+/−^ mice, revealed mildly reduced performance in the grip strength and wire-maneuvering tasks (Table 1). Female *Brd1*^+/−^ mice did not differ significantly on their pain response (Fig. S1A), but were mildly impaired in their motor coordination, as evident from their rotarod performance (Fig. S1B, two-way ANOVA; *p* = 0.047)) and gaiting pattern (Fig. S1F, gaiting uniformity, *t*-test; *p* = 0.039). However, as they performed *at par* with their WT littermates in the beam walking task (Fig. S1G–H), we considered female *Brd1*^+/−^ mice fit for testing in settings assessing complex behaviors. Assessment of motor coordination in male *Brd1*^+/−^ mice has been reported previously (Table S1)^35^.

**Table 1:** Basic neurological functioning and behaviors in female *Brd1*^+/*−*^ mice.

Test	Parameters	Outcome	Implication
Irwin’s observational battery	Undisturbed behavior	—	Basic neurological functioning
	Finger approach	—	Basic neurological functioning
	Touch escape	—	Basic neurological functioning
	Grip strength	↓	Basic neurological functioning
	Visual placing response	—	Basic neurological functioning
	Corneal response	—	Basic neurological functioning
	Toe-pinch response	—	Basic neurological functioning
	Wire-maneuver	↓	Basic neurological functioning
	Limb- and abdominal tone	—	Basic neurological functioning
	Tail-pinch response	—	Basic neurological functioning
Hot-plate	Response	—	Acute pain response
Beam walking	Crossing speed/missteps	—	Motor coordination
Rota-rod	Latency to fall	↓	Motor coordination
Foot-printing test	Stride length	—	Motor coordination
	Base width	—	Motor coordination
	Step uniformity	↓	Motor coordination
Fear Conditioning (FCS)	Conditioning	↓	Conditional learning
	Contextual memory (day 2)	↓	Associative memory*
	Extinction retrieval	—	Associative memory
	Cue dependent learning	—	Associative memory
Acoustic startle reactivity (ASR)	Startle	↑	Hearing/stress susceptibility
	Latency to startle	↓	Stress susceptibility
Prepulse inhibition (PPI)	Baseline	↓	Pre-attentive processing
Locomotor activity	Novelty-induced	—	Psycho-motor activity
	Amphetamine induced	—	Meso-limbic drug responsiveness
	Cocaine-induced	—	Meso-limbic drug responsiveness
8 arm radial maze (ARM)	Re-entry to baited arms#	—	Working memory
	Entry to non-baited arms#	↑	Non-spatial reference memory
Elevated plus maze (EPM)	Time in open arms	—	Anxiety behavior/Mania
Bright open field (BOF)	Time in central zone	—	Anxiety behavior/Mania
Light and dark box (LDB)	Time in light box	—	Anxiety behavior/Mania
Open field test (OF)	Distance moved	—	Anxiety behavior/Mania
Forced swim test (FST)	Immobility	↑	Behavioral despair/Mania
Tail suspension test (TST)	Immobility	↑	Behavioral despair/Mania
Sucrose preference test (SPT)	Sucrose preference	↓	Anhedonia

#**:** number of events. *Likely reflect acquisition deficit during conditioning.

### Locomotor activity and sensorimotor response in *Brd1*^+/−^ mice

**Fig. 1:**
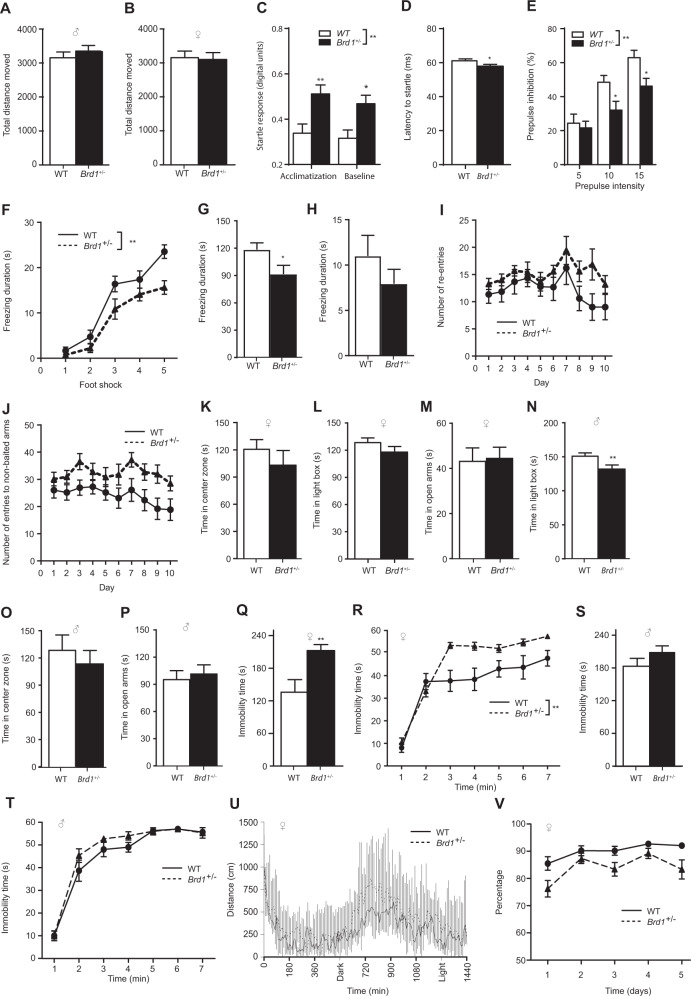
Behavioral characterization in male and female *Brd1*^+/−^ mice. **a** Male mice (Mann–Whitney *U* = 94.0; *p* = 0.46). **b** Female mice (*t* = 0.20; *p* = 0.85): Total distance moved in the open field (OF) (*n* = 15/group). **c** Female *Brd1*^*+/*−^ mice (*n* = 18) displayed significantly increased acoustic startle responsivity (ASR) compared to WT mice (*n* = 17) (genotype effect, *F* = 10.10, *p* = 0.003) both when initially introduced to the test setting (Tukey’s post hoc test, *t* = 3.07; *p* = 0.004) and before baseline PPI testing (Tukey’s post hoc test, *t* = 2.94; *p* = 0.006). **d** Response latency to the startle was furthermore significantly shorter than in WT mice (*t* = 2.09, *p* = 0.044). **e** Female *Brd1*^*+/*−^ mice (*n* = 18) displayed reduced prepulse inhibition (PPI) compared to WT mice (*n* = 17) across the span of tested prepulse intensities (genotype effect, *F* = 4,163, *p* = 0.049); *Cognitive functioning of female Brd1*^*+/*−^*mice*. **f**–**h** Fear conditioning system (FCS) (*n* = 15/group). **f** Time spent as freezing during the repeated presentation of conditioning stimuli (CS; 30 s, 80db, white noise) on day one. Unconditioned stimulus (UCS) was 0.7 mA foot shock for 2 s. Inter-pairing intervals varied with an average of 60 s (30–120 s). Female *Brd1*^*+/−*^ mice displayed significantly less freezing during the conditioning phase of FCS (*F* = 12.26; *p* = 0.002). **g** Duration of freezing behavior within the first 3 min, after entering the conditioned context on the second day. Female *Brd1*^*+/*−^ mice displayed significantly reduced freezing behavior (*t* = 2.23; *p* = 0.03). **h** Time spent as freezing during the first presentation of CS (30 s) in the novel context on the third day. Female *Brd1*^*+/*−^ mice did not display cue-dependent learning deficits. **i**, **j** 8-Arm Radial Maze (8ARM) (*n* = 12/group). **i** Working memory errors, measured by the total number of re-entries to baited arms. **j** Reference memory errors, measured by the total number of entries to non-baited arms. Our 8ARM setup, which provided clear intramaze cues, predominantly assessed non-spatial memory and associative learning. Female *Brd1*^*+/*−^ mice did not show working memory deficits, but displayed significant non-spatial reference memory deficits (*F* = 5.57; *p* = 0.03). **k–m** Female mice. **k** Total time spent in the central zone of bright open field (BOF) (*n* = 15/group). **l** Total time spent in the light box of light and dark box test (LDB) (*n* = 15/group). **m** Total time spent in the open arms of elevated plus maze (EPM) (*n* = 15/group). Female *Brd1*^*+/*−^ mice did not display any anxiety equivalent behaviors. **n**–**p** Male mice. **n** Total time spent in the central zone of BOF (n = 15/group). **o** Total time spent in the light box of LDB (*n* = 15/group). This finding was not corroborated by the BOF and EPM results of male *Brd1*^*+/*−^ mice. **p** Total time spent in the open arms of EPM (*n* = 15/group). Male *Brd1*^*+/*−^ mice spent significantly less time in the light box of LDB (*t* = 2.97; *p* = 0.006), but they did not exhibit similar anxiety-like behaviors in BOF or EPM. **q** Female *Brd1*^*+/*−^ mice were significantly more immobile in TST (*n* = 15/group; *t* = 3.01; *p* = 0.007) (**r**) and in FST (*n* = 15/group; *F* = 12.26; *p* = 0.002). **s** Male *Brd1*^*+/*−^ mice did not display such behavioral despair in TST (*n* = 15/group) (**t**) and FST (*n* = 15/group). **u** Female *Brd1*^+/−^ mice did not differ from WT mice in distance moved over 24 h (*n* = 10/group). Dark indicates the time when the lights were switched off in the stable, while light indicates the time when they were switched on. **v** Female *Brd1*^+/−^ mice showed significantly less sucrose preference than the WT mice (*n* = 11/group; *F* = 14.03; *p* = 0.001), sucrose preference (weight of 2% sucrose solution consumed/ weight of total fluid consumed) in percentage. Data shown are mean and SEM for each group *p < 0.05; **p < 0.01.

General locomotor activity was assessed in the OF where male (Fig. 1a) and female (Fig. 1b) *Brd1*^+/−^ mice performed *at par* with their WT littermates. As reported in male *Brd1*^+/−^ mice (Table S1)^35^, female *Brd1*^+/−^ mice displayed significantly increased acoustic startle responsivity (ASR) (Fig. 1c, two-way ANOVA; *p* = 0.003), both when initially introduced to the test setting (Fig. 1c, Tukey’s post hoc test; *p* = 0.004) and before baseline PPI testing (Fig. 1c, Tukey’s post hoc test; *p* = 0.006). Response latency to the startle was furthermore significantly shorter in female *Brd1*^+/−^ mice than in WT mice (Fig. 1d, *t*-test; *p* = 0.044). The magnitude of baseline startle is known to influence PPI^43^. Accordingly, female *Brd1*^+/−^ mice displayed reduced PPI across the span of tested prepulse intensities (Fig. 1e, two-way ANOVA; *p* = 0.049).

### Cognition

Female *Brd1*^+/−^ mice froze significantly less than WT mice during the conditioning phase of FCS (Fig. 1f, two-way ANOVA; *p* = 0.002) and when returning to the same context on the following day (Fig. 1g, *t*-test; *p* = 0.03), collectively suggestive of a central acquisition deficit in female *Brd1*^+/−^ mice. However, female *Brd*1^+/−^ mice did not differ significantly from the WT mice on their cue dependent learning (Fig. 1h and Table 1) or on working memory errors in 8ARM (Fig. 1i). They did, however, make significantly more entries into the never-baited arms, indicative of impaired reference memory (Fig. 1j, two-way ANOVA; *p* = 0.03) (Table 1). Cognitive performance of male *Brd1*^+/−^ mice tested in parallel has previously been reported^35,36^ and results are summarized in Table S1.

### Affective behaviors in *Brd1*^+/−^ mice

Affective behaviors were assessed in both male and female *Brd1*^+/−^ mice (Table 1 and Table S1). Female *Brd1*^+/−^ mice did not differ significantly in the time spent in the central zone of BOF (Fig. 1k), in the light box of LDB (Fig. 1l) or in the open arms of EPM (Fig. 1m). Although male *Brd1*^+/−^ mice spent significantly less time in the light box of LDB (Fig. 1n, *t-*test; *p* = 0.006), they did not exhibit similar anxiety-like behaviors in BOF (Fig. 1o) or EPM (Fig. 1p). Suggestive of behavioral despair, female *Brd1*^+/−^ mice were significantly more immobile in TST (Fig. 1q, *t*-test; *p* = 0.007) and in FST (Fig. 1r, two-way ANOVA; *p* = 0.002) compared to WT mice, whereas this was not evident in male *Brd*1^+/−^ mice (Fig. 1s, t). FST male WT immobility was higher than in female WT mice (Wilcoxon rank-sum; *p* = 0.001) and the WT male and female mice did not differ significantly on their TST immobility (*t*-test; *p* = 0.09)(Fig. 1q–t). Both TST (two-way ANOVA; *p* = 0.013) and FST (two-level mixed effects GLM; *p* = 0.014) data confirmed statistically significant interactions between gender and genotypes. Hence, we decided to assess additional affective behaviors in female mice only. Circadian rhythm, measured as 24HLM performance, appeared unaltered in female *Brd1*^+/−^ mice (Fig. 1u) whereas sucrose preference was significantly reduced in female *Brd1*^+/−^ mice compared to WT mice (Fig. 1v, two-way ANOVA; *p* = 0.001).

### Neurochemistry and psychotropic drug-induced activity

**Fig. 2:**
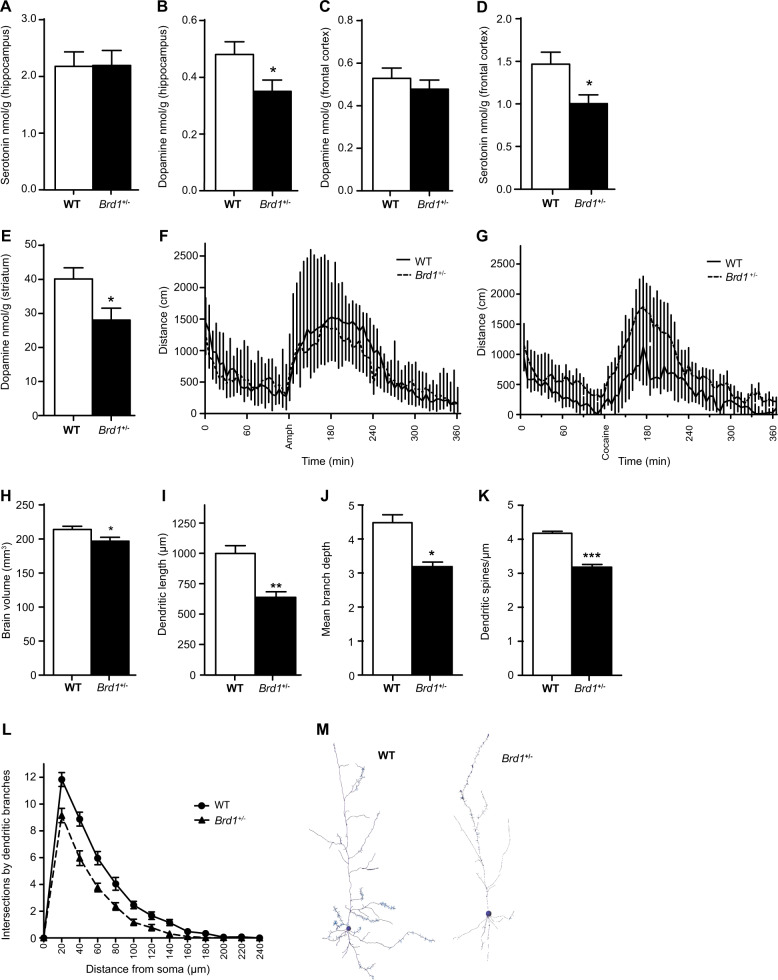
Neurochemistry and psychotomimetic drug sensitivity in female *Brd1*^+/−^ mice. **a**–**e** Neurotransmitter levels were determined by HPLC in several brain tissues in female *Brd1*^+/−^ mice in parallel with male *Brd1*^+/−^ mice as previously reported on ref. ^35^. **a** Female mice displayed unaltered hippocampal serotonin level (*n* = 9/group). **b** Significantly reduced hippocampal dopamine level (*n* = 10/group, *t* = 2.147, *p* = 0.045). **c** Unaltered fronto-cortical dopamine (*n* = 9/group). **d** Less fronto-cortical serotonin (*n* = 15/group; *t* = 2.70; *p* = 0.01) and; **e** reduced striatal dopamine (*n* = 10/group; *t* = 2.52; *p* = 0.02) compared to WT mice. 5-HT: 5-hydroxy tryptamine (serotonin); DA: dopamine. **f** Distance moved before and after amphetamine 5 mg/kg (Amph) injection was similar in female *Brd1*^+/−^ and WT mice (*n* = 10/group). **g** Distance moved before and after cocaine 30 mg/kg injection was similar in female *Brd1*^+/−^ and WT mice (*n* = 12/group). **h** Total brain volume was slightly reduced in female *Brd1*^+/−^ mice compared to WT mice (*n* = 7/group; *t* = 2.31; *p* = 0.041). **i** Total dendritic length including apical and basal dendrites. aCC pyramidal neurons had significantly shorter dendrites in female *Brd1*^+/−^ mice compared to WT mice (*t* = 3.29; *p* = 0.008). **j** Mean branch depth: branching depth was defined by the number of bifurcations from the beginning point to the end of a dendrite. Female *Brd1*^+/−^ mice had less dendritic branching (*t* = 3.08; *p* = 0.01) compared to WT mice. **k** Mean dendritic spine density (number of spines/ length of dendrites). Female *Brd1*^+/−^ mice had less dendritic spine density (*t* = 9.19; *p* < 0.001) compared to WT mice. **l** 3-D Sholl analysis: Number of dendritic intersections on concentric spheres (radius interval 20 µm) with their centres at soma. Neurons in female *Brd1*^+/−^ mice had significantly less dendritic branching (*F* = 20.60; *p* < 0.001) than neurons in WT mice. **m** 3-D reconstruction of left: WT neuron and right: *Brd1*^+/−^ neuron. Data shown are mean and SEM for each group *p < 0.05; **p < 0.01; ***p < 0.001.

As reported in male *Brd1*^+/−^ mice (Table S1)^35^, female mice displayed unaltered hippocampal serotonin levels (Fig. 2a) and significantly reduced hippocampal dopamine levels (Fig. 2b, *t*-test; *p* = 0.045) but unaltered fronto-cortical dopamine levels (Fig. 2c). However, female mice had significantly less fronto-cortical serotonin (Fig. 2d, t-test; *p* = 0.01) and, noticeably, significantly reduced striatal dopamine (Fig. 2e, *t*-test; *p* = 0.02) compared to WT mice. Further analyses confirmed statistically significant interactions between female gender and *Brd1*^+/−^ genotype on fronto-cortical serotonin (two-way ANOVA; *p* = 0.002) and striatal dopamine (two-way ANOVA; *p* = 0.002) levels. Additionally, their sensitivity towards the psychomotor stimulatory effects of amphetamine 5 mg/kg (Fig. 2f) and cocaine 15 or 30 mg/kg (Fig. 2g) did not differ from the sensitivity of WT mice.

### Brain volume and neuronal morphology

Total brain volume, as estimated by stereology, was slightly reduced (~8%) in female *Brd1*^+/−^ mice (Fig. 2h and Fig. S1A, *t*-test; *p* = 0.041), but with no difference in brain symmetry (Fig. S2B) or ventricle volume (Fig. S2C, D). In line with reduced overall brain tissue volume, aCC pyramidal neurons had significantly shorter dendrites in female *Brd1*^+/−^ mice compared to WT mice (Fig. 2i, *t*-test; *p* = 0.008) combined with less dendritic branching (Fig. 2j, *t*-test; *p* = 0.01) and less dendritic spine density (Fig. 2k, *t*-test; *p* < 0.001). 3-D Sholl analysis counting the dendritic intersections on the concentric spheres with their centres at soma confirmed that these neurons had significantly less dendritic branching (Fig. 2l, m, *p* < 0.001).

### Behavioral response to antidepressants

**Fig. 3:**
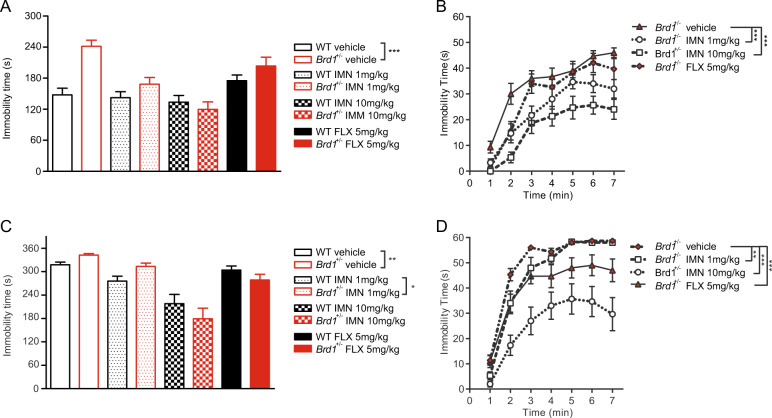
Behavioral response to antidepressant medication in female *Brd1*^+/−^ mice. **a**, **b** Tail suspension test. **a** Total duration of immobility in the 8 groups (*n* = 15/group). Comparison between vehicle-treated WT and vehicle-treated *Brd1*^*+*^^*/−*^ mice confirmed the depression-like behaviors of female *Brd1*^*+/−*^ mice (*t* = 5.42; *p* < 0.001). Comparisons between other three pairs indicated that the depressive phenotype of female *Brd1*^*+/−*^ mice could be reversed by antidepressant medications. **b** Duration of immobility in the four groups of female *Brd1*^*+/−*^ mice, which received four different interventions. Female *Brd1*^*+/−*^ mice, that received imipramine 1 (*F* = 17.55; *p* < 0.001) and 10 (*F* = 43.08; *p* < 0.001) mg/kg, displayed significantly less depression-like behaviors than the vehicle-treated *Brd1*^*+/−*^ mice. **c**, **d** Forced swim test. **c** Total duration of immobility in the 8 groups (*n* = 15/group). Comparison between vehicle-treated WT mice and vehicle-treated *Brd1*^*+/−*^ mice confirmed the depression-like behaviors of female *Brd1*^*+/−*^ mice (*t* = 3.14; *p* = 0.005). Except for 1 mg/kg IMN treated groups, comparisons between other two pairs indicated that the depressive phenotype of female *Brd1*^*+/−*^ mice could be reversed by antidepressant medications. **d** Duration of immobility in the four groups of female *Brd1*^*+/−*^ mice, which received four different interventions. Female *Brd1*^*+/−*^ mice that received imipramine 1 (*F* = 9.69; *p* = 0.004) and 10 (*F* = 35.71; *p* < 0.001) mg/kg, and FLX 5 mg/kg (*F* = 18.05; *p* < 0.001) displayed significantly less depression-like behaviors than the vehicle-treated *Brd1*^*+/−*^ mice. IMN, imipramine; FLX, fluoxetine. Data shown are mean and SEM for each group. *p < 0.05; **p < 0.01; ***p < 0.001.

Provided that the phenomenological and pathophysiological phenotype of female *Brd1*^+/−^ mice indicate translational relevance to depressive disorders, they may be reversible upon the administration of clinically used antidepressants. In accordance, treatment with Fluoxetine (FLX) and Imipramine (IMN) reversed the despair-like behaviors of female *Brd1*^+/−^ mice, while having negligible effect on their movements in the OF (Fig. S3). Particularly, differences in immobility between *Brd1*^+/−^ and WT mice during TST, could be reversed by IMN 1 (Fig. 3a) or 10 mg/kg (Fig. 3a), and by FLX 5 mg/kg (Fig. 3a) with *Brd1*^+/−^ mice receiving IMN (Fig. 3b; 1 mg/kg, two-way ANOVA; *p* < 0.001 or 10 mg/kg, two-way ANOVA; *p* < 0.001) being significantly less immobile during TST than the vehicle-treated *Brd1*^+/−^ mice. Similarly, the differences in immobility during FST, could be reversed by IMN 10 mg/kg (Fig. 3c) and by FLX 5 mg/kg (Fig. 3c), with IMN (Fig. 3d, two-way ANOVA; *p* < 0.001) or FLX (Fig. 3d, two-way ANOVA; *p* < 0.001) causing reduced immobility compared to vehicle-treatment. Vehicle-treated *Brd1*^+/−^ mice were significantly more immobile in TST (Fig. 3a, *t*-test; *p* < 0.001) and FST (Fig. 3c, *t*-test; *p* = 0.005) than the vehicle-treated WT mice thus replicating the behavioral despair in untreated female *Brd1*^+/−^ mice.

### Global gene expression profiling of antidepressant treatment in *Brd1*^+/−^ mice

**Fig. 4:**
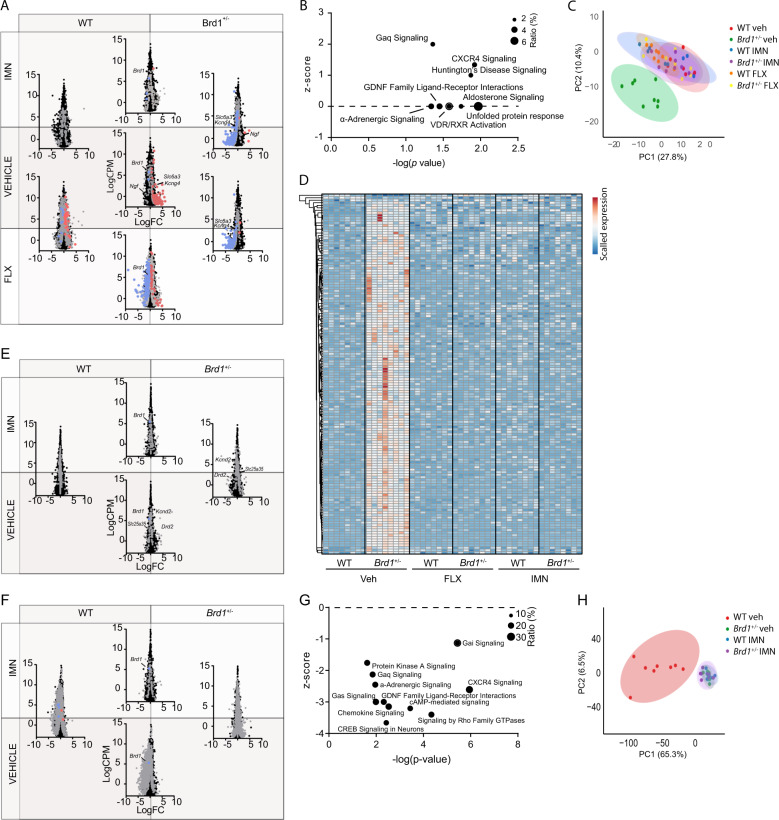
Cerebral gene expression profiling of cortical and subcortical brain tissues in vehicle and antidepressant administered female *Brd1*^+/−^ mice. **a** Gene expression profile in AMG tissue from vehicle, IMN and FLX administered female WT (*n* = 8/group) and *Brd1*^+/*−*^ (*n* = 8/group) mice. Gray, red, and blue color mark genes that are nominally significantly differentially expressed, significantly upregulated after FDR correction, and significantly downregulated after FDR correction between *Brd1*^+/*−*^ and WT mice, respectively. **b** Functional genomic analysis using ingenuity pathway analysis IPA software identifies pathways that are enriched with nominally significantly AMG DEGs and predicts an activation score (*z*-score) based on the direction of regulation (up or down) of DEGs. Diameter of circles represent the overlap between DEGs and genes annotated to the respective pathways. **c** Principal component analysis of all genes expressed (CPM > 0.1) in each sample, cluster vehicle administered *Brd1*^+/*−*^ mice separately from vehicle WT mice, and shows that treatment with IMN or FLX normalizes amygdaloid gene expression in *Brd1*^+/*−*^ mice. **d** Heatmap showing the 144 genes surviving correction for multiple testing (FDR). DEGs are generally more abundantly expressed in vehicle administered *Brd1*^+/*−*^ mice than in vehicle administered WT mice, whereas IMN or FLX both completely normalizes expression of this subset of genes. **e** Gene expression profile in aCC tissue from vehicle administered and IMN administered female WT (*n* = 8–10/group) and *Brd1*^+/*−*^ (*n* = 8–10/group) mice. Gray color marks genes that are nominally significantly differentially expressed between *Brd1*^+/*−*^ and WT mice. **f** Gene expression profile in CPu tissue from vehicle administered and IMN administered female WT (*n* = 8–10/group) and *Brd1*^+/*−*^ (*n* = 8–10/group) mice. Gray, red, and blue color mark genes that are nominally significantly differentially expressed, significantly upregulated after FDR correction, and significantly downregulated after FDR correction between *Brd1*^+/*−*^ and WT mice, respectively. **g** Functional genomic analysis using ingenuity pathway analysis IPA software identifies pathways that are enriched with nominally significantly CPu DEGs and predicts an activation score (*z*-score) based on the direction of regulation (up or down) of DEGs. Diameter of circles represent the overlap between DEGs and genes annotated to the respective pathways. **h** Principal component analysis of all genes expressed (CPM > 0.1) in each sample, cluster vehicle administered *Brd1*^+/*−*^ mice separately from vehicle administered WT mice but shows that CPu treatment effect of IMN, in terms of gene expression changes, is largely on WT mice and not *Brd1*^+/*−*^ mice.

To delineate the molecular signatures accompanying the behavioral response to antidepressants by *Brd1*^+/−^ mice, we conducted global gene expression profiling of selected brain tissues from mice administered vehicle or antidepressant (IMN or FLX). Amygdaloid (AMG) tissue from vehicle administered mice was characterized by pronounced changes in gene expression involving 144 differentially expressed genes (DEGs) that were significant after Benjamini–Hochberg false discovery rate (FDR) correction at 5% (Fig. 4a (mid panel) and Table S2). As these comprised a high number of predicted/uncharacterized genes (Table S2) we subjected nominally significant DEGs (*p* < 0.01, 511 genes) to functional analyses using ingenuity pathway analysis (IPA) software. This revealed an overrepresentation of genes acting in biological pathways previously implicated with affective behaviors, including unfolded protein response^44^ and α-adrenergic^45^-, chemokine^46^-, G-protein coupled receptor (GPCR), and Glial cell-derived neurotrophic factor (GDNF) mediated signaling^47^ (Fig. 4b and Table S3). Notably, fold change of implicated DEGs suggested an activation of G-protein coupled receptor (GPCR) mediated signaling, specifically through the Gaq subunit associated with, among others, the 5HT_2_ serotonergic-, and Alpha-1 adrenergic receptors (Fig. 4b and Table S3). Supportive of altered amygdaloid intracellular signaling in *Brd1*^+/−^ mice, predicted upstream DEG regulators comprised a range of stimulus-induced transcription factors, including FOS, CREB1 and, ADORA2A (Table S4).

Overall, administration of FLX and IMN normalized the amygdaloid transcriptome in *Brd1*^+/−^ mice (Fig. 4c), and, convincingly, FLX and IMN administration nearly completely normalized AMG expression of genes differentially expressed in vehicle administered *Brd1*^+/−^ mice (Fig. 4d and Table S6 and S6) without affecting *Brd1* expression (Fig. 4a). Whereas the tested dose (10 mg/kg) of IMN had only minor effect on AMG gene expression in WT mice, administration of FLX (5 mg/kg) resulted in massive changes in WT mice (Fig. 4a). Accordingly, we proceeded with testing only IMN (10 mg/kg) in tissue samples from aCC and striatum, caudate putamen (CPu). In aCC, only *Brd1* was significantly differentially regulated after FDR correction between *Brd1*^+/−^ and WT vehicle groups (Fig. 4e, lower panel) and only 141 genes were differentially expressed at a nominal significant cut-off set at 1% (Table S7). This set of genes did not cluster in distinct biological pathways (Table S8). Like in AMG, however, administration of antidepressants primarily affected gene expression in *Brd1*^+/−^, and to a lesser extent, WT mice (Table S9). This included a reduction in mRNA encoding dopamine receptor 2 (*Drd2*, Fig. 4e and Table S9), which was significantly overexpressed in vehicle administered *Brd1*^+/−^ mice compared to WT mice (Fig. 4e and Table S7). In CPu, only *Brd1* was significantly downregulated after FDR correction in the vehicle treated group, (Fig. 4f, lower panel and Table S10) whereas a large number of genes (2260) were differentially expressed at a nominally significant cut-off set at 1% (Fig. 4f and Table S10). Although CPu and AMG DEGs generally clustered in the same functional pathways and involved same predicted upstream transcriptional regulators (Tables S2, S11, 12), contrary to AMG DEGs, CPu DEGs consistently reported of inhibited intracellular signaling initiated by GPCR subunits associated with a broad range of neurotransmitter receptors (Fig. 4g and Table S11). Although treatment with IMN overall normalized CPu gene expression between *Brd1*^+/−^ and WT mice (Fig. 4f (Top panel)), effects were more pronounced in WT (Table S13, 1370 nominally significant DEGs) than in *Brd1*^+/−^ mice (Table S14, 513 nominally significant DEGs) (Fig. 4h).

## Discussion

Despite the clinical heterogeneity of affective disorders, their, etiopathologies are partially overlapping and include an intricate interplay between environmental, genetic, and epigenetic factors^1,8,48–50^. Post translational modifications of histones, such as Kac, have been linked with brain development^7^ and particularly the pathophysiology of affective disorders^8,51^. Acting as an epigenetic regulator during neurodevelopment, BRD1 has the potential to integrate intrinsic and environmental signals into the shaping of the maturing brain. Here, we demonstrate that reduced *Brd1* expression in female mice results in brain morphometric alterations accompanied by changes in behaviors and underlying neurobiology with broad translational relevance to affective disorders. Supporting the predictive validity of female *Brd1*^+/−^ mice as a model for depressive pathology, behavioral and molecular changes in *Brd1*^+/−^ mice are reversible upon the administration of clinically effective antidepressants. Finally, integrative genomic profiling of regional brain transcriptomic effects reveals molecular mechanisms and tissue specificity associated with affective pathology and antidepressant treatment effect.

### Neuro-implication of reduced *Brd1* expression is sex-biased

Cognitive impairments are common in psychiatric disorders, and although more thoroughly investigated in male *Brd1*^+/−^ mice^36^, both sexes display cognitive impairments with broad translational relevance, including central acquisition deficits and impaired reference memory. Sensorimotor deficits in the form of increased baseline startle amplitude, which have been reported in a range of psychiatric disorders^52^, are similarly seen in both male and female *Brd1*^+/−^ mice. However, neither male nor female *Brd1*^+/−^ mice display consistent changes in their risk-taking behaviors. Female *Brd1*^+/−^ mice, additionally, did not exhibit marked changes in their circadian cycle as measured by 24HLM. However, supporting their translational value as model of depressive symptomatology seen in affective disorders^53^ and the prodromal stage of schizophrenia^54^, female *Brd1*^+/−^ mice displayed increased immobility during FST and TST indicating behavioral despair^55^, and decreased sucrose preference representing anhedonia^56^.

Similar to what has been reported in both schizophrenia, bipolar disorder^57^, and depressed suicide victims^58^, female *Brd1*^+/−^ mice display abnormal brain and neuronal morphology with reduced dendritic branching and spine pathology^38^. Although these parameters have not been assessed in male *Brd1*^+/−^ mice, detailed structural brain imaging followed by stereological estimation of regional volumes and cell number, have revealed reduced subcortical volume and striatal cell loss in male *Brd1*^+/−^ mice^37^. Sex differences in animal models of psychiatric disorders are, however, common and may mirror the documented sex differences in psychiatric disorders where symptom profiles and severity differ between sexes^59–64^ and where depressive disorders are more prevalent among women than men^59,63^. In line with the reported divergences in behaviors, the neurochemical profile of female *Brd1*^+/−^ mice varied significantly from what we have previously reported in male *Brd1*^+/−^ mice^35,36^. Unlike male *Brd1*^+/−^ mice^35^, female *Brd1*^+/−^ mice are not super-sensitive to the psycho-motor stimulatory effect of cocaine and PCP. Although both male and female mice display increased hippocampal dopamine, only female *Brd1*^+/−^ mice displayed significantly reduced levels of cortical serotonin and striatal dopamine, consistent with the monoamine hypothesis of depression^65^. Histone modifications act to epigenetically sexually differentiate the developing brain and consequently behavior^66^ by regulating the genomic actions of sex steroid hormones^66,67^. Intriguing in this context, BRD1 is a co-regulator of nuclear hormone receptor-mediated signaling^17,18,23^ and its chromatin interactome enriched with estrogen and androgen target genes^17^.

### Neuro-molecular effect of reduced *Brd1* expression reflect brain regional changes in intracellular signaling activity

Using transcriptomic profiling to broadly capture Brd1-mediated neuro-molecular changes across multiple brain regions, we find that the dopamine receptor 2 (*Drd2*) and dopamine transporter (*Slc6a3*) are among the dysregulated genes in aCC and AMG, respectively (Fig. 4a, e). Monoamines act through activation of either G protein or ion channel linked surface receptors, which trigger second messenger systems (e.g. 3′–5′-cyclic adenosine monophosphate (cAMP), Rho family GTPases, inositol 1,4,5-trisphosphate (IP3) or calcium (Ca^2+^)). They, in turn, activate downstream kinases (e.g. protein kinase A (PKA)), which phosphorylate the transcription factor cAMP response element-binding protein (CREB) that regulates expression of many immediate early and late response genes. Convincingly, DEGs across examined tissues particularly cluster in GPCR-controlled intracellular signaling pathways and comprise immediate early and late response genes like neurotrophic factors (e.g. GDNF^68^ and NGF). Dysregulation of these signaling cascades are commonly reported in affective disorders^69–73^, reduced levels of phosphorylated CREB^74^ and neurotrophin receptors^75^ have been reported in the post-mortem frontal cortices of people with MDD and cortical IP3 has been suggested as a biomarker for depressive symptoms across diagnostic boundaries^76^. Interestingly, DEGs in the examined brain tissues reflect regional differences in intracellular signaling activity. Particularly, transcriptomic data from AMG tissue suggest increased Gaq mediated signaling, and thus increased PI3 activity, whereas this is the opposite in CPu tissue. CPu gene expression was further associated with reduced signaling mediated by Gas and Gai and their associated second messenger (cAMP).

### The antagonistic effect of antidepressants on BRD1 mediated dysregulation is tissue specific

Supporting the predictive validity of *Brd1*^+/−^ mice as a model for depressive psychopathology, administration of either of the two tested antidepressants, IMN and FLX, effectively alleviated the behavioral changes displayed by *Brd1*^+/−^ mice in the TST and FST without affecting basal motor behaviors. This effect was further apparent at the molecular level, where IMN administration essentially normalized AMG gene expression without affecting the expression of *Brd1*. This was also the case for FLX, but at the selected dose, significant gene-regulatory changes were additionally seen in WT mice. In aCC, IMN treatment completely normalized *Drd2* expression and *Slc6a3*, possibly reflecting a normalization of dopamine signaling in this tissue. Furthermore, expression of *Slc25a35*, which is a marker of mitochondrial dysfunction in brain regions under experimental mixed anxiety/depression-like disorder^77^, was additionally normalized following IMN administration (Fig. 4e). However, as basal gene expression, measured in the vehicle administered group, did not differ much between *Brd1*^+/−^ and WT mice, the neuromolecular effect of antidepressant treatment was less apparent in this tissue. Surprisingly, in CPu, where large changes in basal gene expression were observed between WT and *Brd1*^+/−^ mice, the effect IMN administration was much more pronounced in WT than in *Brd1*^+/−^ mice. Similar findings have been reported from other rodent models with depressive-like phenotypes^78,79^.

Histone modification and chromatin modeling play a role in multiple physiological and pathological processes in the brain, including cognition^80^, circadian rhythms^81^, and the development of affective pathology^8,51^. Here we show that the schizophrenia and bipolar disorder associated epigenetic reader, BRD1, governs affective behaviors and associated neuromolecular and biological pathways in mice. In line with BRD1’s reported function as a co-regulator of nuclear hormone receptors^17,18,23^ and their targeted transcriptional response to gonadosteroid and corticosteroid signaling^17^, the effect of reduced *Brd1* expression is sex-biased, with only female mice displaying changes in affective behaviors. *BRD1* may thus provide an important link between averse environmental risk factors (.e.g. sex and stress) and depression. While equating *Brd1* deficiency with depression susceptibility is over-simplistic, female *Brd1*^+/−^ mice set the stage for further studies evaluating the epigenetic changes and neurodevelopmental abnormalities, pertinent to depression.

## Supplementary information

Supplementary Information

Supplementary Tables

## Data Availability

All raw and processed sequencing data have been deposited in NCBI’s Gene Expression Omnibus^82^ and are accessible through GEO Series accession number GSE150265.
