# Supplementary material for: Reduced Brd1 expression leads to reversible depression-like behaviors and gene-expression changes in female mice
Source: Transl Psychiatry. 2020 Jul 17;10:239. doi: 10.1038/s41398-020-00914-2 (PMC7367888; doi:10.1038/s41398-020-00914-2)
Supplement: Supplementary file 1 — Supplementary Information [file 41398_2020_914_MOESM1_ESM.docx]

**Reduced *Brd1* expression leads to reversible depression-like behaviors and**

**gene-expression changes in female mice**

Supplemental Information

Contents

[Supplemental Methods and Materials 3](#_Toc39178675)

[*Animals* 3](#_Toc39178676)

[*Functional observation battery (HLu)* 3](#_Toc39178677)

[*Acute pain response (HLu)* 4](#_Toc39178678)

[*Rotarod (AU)* 4](#_Toc39178679)

[*Balance beam walking (AU)* 4](#_Toc39178680)

[*Foot printing (AU)* 4](#_Toc39178681)

[*Open Field test (OF - AU))* 5](#_Toc39178682)

[*Bright Open Field test (BOF - AU)* 6](#_Toc39178683)

[*Light and dark box (LDB - AU)* 6](#_Toc39178684)

[*Elevated Plus Maze (EPM - AU)* 6](#_Toc39178685)

[*Fear Conditioning System (FCS - AU)* 6](#_Toc39178686)

[*8-Arm Radial Maze (8ARM - AU)* 6](#_Toc39178687)

[*24 Hours Locomotion (24HLM - AU)* 7](#_Toc39178688)

[*Prepulse inhibition (HLu)* 7](#_Toc39178689)

[A*mphetamine (AIH) or Cocaine Induced Hyperactivity (CIH) (AU)* 7](#_Toc39178690)

[*High-Performance Liquid Chromatography (HPLC)* 7](#_Toc39178691)

[*Estimation of brain volumes* 7](#_Toc39178692)

[Supplemental Tables 9](#_Toc39178693)

[Table S1: Basic neurological functioning and behaviors in male and female *Brd1*^+/-^ mice 9](#_Toc39178694)

[Table S2: AMG DEG analysis of female *Brd1*^+/-^ vehicle vs wild type vehicle 10](#_Toc39178695)

[Table S3: Canonical IPA analysis of AMG DEGs between female *Brd1*^+/-^ vehicle vs wild type vehicle 10](#_Toc39178696)

[Table S4: Upstream IPA analysis of AMG DEGs between female *Brd1*^+/-^ vehicle vs wild type vehicle 10](#_Toc39178697)

[Table S5: DEG analysis of AMG DEGs between female *Brd1*^+/-^ FLX vs *Brd1*^+/-^ vehicle 11](#_Toc39178698)

[Table S6: AMG DEG analysis of female *Brd1*^+/-^ IMN vs *Brd1*^+/-^ vehicle 11](#_Toc39178699)

[Table S7: aCC DEG analysis of female *Brd1*^+/-^ vehicle vs wild type vehicle 11](#_Toc39178700)

[Table S8: Canonical IPA analysis of aCC DEGs between female *Brd1*^+/-^ vehicle vs wild type vehicle 11](#_Toc39178701)

[Table S9: aCC DEG analysis of female *Brd1*^+/-^ IMN vs *Brd1*^+/-^ vehicle 11](#_Toc39178702)

[Table S10: CPu DEG analysis of female *Brd1*^+/-^ vehicle vs wild type vehicle 11](#_Toc39178703)

[Table S11: Canonical IPA analysis of CPu DEGs between female *Brd1*^+/-^ vehicle vs wild type vehicle 11](#_Toc39178704)

[Table S12: Upstream IPA analysis of CPu DEGs between female *Brd1*^+/-^ vehicle vs wild type vehicle 11](#_Toc39178705)

[Table S13: CPu DEG analysis of female wild type IMN vs wild type vehicle 11](#_Toc39178706)

[Table S14: CPu DEG analysis of female *Brd1*^+/-^ IMN vs *Brd1*^+/-^ vehicle 11](#_Toc39178707)

[Supplemental Figures 12](#_Toc39178708)

[Figure S1.Basic neurological functioning and motor coordination 12](#_Toc39178709)

[Figure S2. Brain and neuron morphometry in *Brd1*^+/-^ mice. 14](#_Toc39178710)

[Figure S3. Open field test on *Brd1*^+/-^ and WT mice following injection with vehicle, IMN or FLX. 15](#_Toc39178711)

[Supplemental References 16](#_Toc39178712)

# Supplemental Methods and Materials

*Animals****:*** A mouse line heterozygous for a targeted deletion in the *Brd1* gene was generated by TaconicArtemis GmbH (Cologne, Germany) C57BL/6NTac-Brd1tm1.2Arte/AborgMmucd (*Brd1*^+/-^) as previously described ^1^. The mouse strain has been deposited and is available at Mutant Mouse Resource and Research Center (MMRRC) at University of California at Davis (RRID:MMRRC_065563-UCD). Mice were bred and housed until the age of 6 weeks at Taconic MB A/S (Lille Skensved, Denmark) and tail biopsies were collected for Polymerase Chain Reaction (PCR) based genotyping after weaning (P21). For tests, conducted at the facilities of H. Lundbeck A/S (HLu), 4-8 *Brd1*^+/-^ and wild type (WT) mice were group housed under controlled laboratory conditions (temperature:21±2°C; humidity:55±5%) for a minimum of one week before behavioral testing. For tests, conducted at the facilities of Aarhus University (AU), mice were group housed 2–6 mice per cage and maintained on 23±2°C temperature. Mice were kept on a 12:12h light–dark cycle. Mice were housed in Macrolon (type II) cages with standard sawdust bedding and standard rodent food and tap water available *ad libitum* (unless stated differently). The cages were enriched with igloos, wooden chew blocks and paper for nesting.

*Functional observation battery (HLu)****:*** Mice between 56-63 days of age (young adults) were initially observed in a transparent observation box (20 x 25 x 14 cm) for any signs of abnormal behavior as described by Irwin ^2^. The following responses were tested: grip-strength, visual placing response, wire-maneuver, corneal responses, and reactivity to pain induced by pinching (toes and tail). Behavioral scoring was performed blinded by a trained experimenter and each behavioral measure was scored using a point system: 0—normal behavior, 1—mild to moderate impairment, and 2— severe impairment. Behaviors were grouped as follows: 1) Sensorimotor function included measures of vision (visual placing response, corneal reflex) and pain (tail pinch and toe pinch); 2) Fear responses included avoidance-escape behaviors (touch escape, finger approach) and other fear behaviors (freezing, difficult handling); 3) Neuromuscular function such as muscle tone (grip-strength, abdominal and limb tone) and motor coordination (wire-maneuver, body posture, and walking); 4) Bizarre behaviors; and 5) Tremors and convulsions. The total score for each group of behavior was compared between genotypes.

*Acute pain response (HLu)****:*** Mice were brought to the testing room and allowed to acclimatize for 10 min. before the test. Pain reflexes in response to a thermal stimulus were measured using a Hot Plate (25.4 x 25.4 cm), surrounded by a clear acrylic cage (19 cm tall, open top). Surface of the plate was heated to a constant temperature of 55°C, as measured by a built-in digital thermometer. Mice were then placed on the hot plate, and the Start/Stop button on the timer was activated. The latency to respond with either a hind paw lick, hind paw flick, or jump (whichever came first) was measured to the nearest 0.1 sec. by deactivating the timer once a response was observed. Mice were moved from the hot plate and returned to its home cage immediately after first sign of discomfort. If a mouse did not respond within 30 sec., the test was terminated and a delay of 30 sec. was noted for the given mouse. Animals were tested one at a time and they were not habituated to the apparatus prior to testing. Each animal was tested in 3 trials with a 20 min. intertrial rest period. The hotplate was cleaned between animals and was allowed to stabilize at test temperature.

*Rotarod (AU)****:*** Rotarod analysis was performed on a 47600 ROTA-ROD with a 30 mm rod (Ugo Basile, Varese, Italy), with two consecutive sessions of three trials per day for three consecutive days. Mice were allowed to rest for 20 min. between trials and sessions were separated by a 6 hours rest period. The rotarod was set to accelerate from 4-40 rpm over a 5-minute period, followed by an additional 5 min. at 40 rpm. Both maximum and average latencies to fall off the apparatus were scored for each session.

*Balance beam walking (AU)****:*** Mice were trained to walk along a balance beam (1 cm wide and 80 cm long) suspended 30 cm above the ground to an enclosed platform at the other end. Two trials were performed for each mouse each day for three consecutive days with 20 min. rest between trials. The time required to cross the beam from start to end (latency) was evaluated and the number of hind-foot missteps was counted. The better of two trials was scored.

*Foot printing (AU)****:*** A 60 x 10 x 10 cm track was constructed with a semi-open upper structure that left the runway in partial shade. Mice were trained to run from the start of the track to the box without stopping. As mice preferred the dimed side of the track, they would run in a straight line in an unconstrained manner. At the time of testing, different colors of water-based ink was applied to front- and hind paws, respectively. Footprints were analyzed for 4 parameters: 1) Stride length: Average distance of forward movement between each stride; 2) Hind-base width and 3) front-base width: Measured as the average distance between left and right hind footprints and left and right front footprints, respectively; and 4) Front/hind paw footprint overlap, a measure of uniformity of step alternation. Latency to cross the track was recorded to ensure that mice were equally motivated to reach the box.

***Forced Swim Test (FST - AU)***: Mice were placed individually in acrylic plastic cylinders (25 cm height×10 cm diameter), filled with water (25°C) to the height of 10 cm, for 7 min. Video recordings were scored manually for displaying immobility, swimming, or climbing every 5 sec. Immobility was defined as the absence of any movements except the minimum essential movements to remain afloat. Swimming was defined as any horizontal movement involving at least two limbs. Climbing was defined as any vertical movement during which their front paws touched the sides of the cylinder ^3^.

***Tail Suspension Test (TST - AU)***: Mice were suspended for 7 min. on the horizontal hooks within test chambers 30 cm above the floor by adhesive tape, placed one cm from the tip of their tails. Video recordings were scored manually for displaying immobility or struggle every 5 sec. Immobility was defined as the absence of any movements in their body and four limbs ^4^.

***Sucrose Preference Test (SPT - AU)***: Mice were single-housed for a week, and then habituated to have two bottles (one with water and another with 2% sucrose solution) on their home-cages for three days. Over the next 5 days, both bottles were weighed, and their positions were switched daily. Sucrose preference was calculated as the ratio between sucrose consumption and total fluid consumption ^5^.

*Open Field test (OF - AU))*: Mice were placed individually in an open field (60×60×30 cm), illuminated at 15 lux, for 5 min.. Video-recorded movements were analyzed by Ethovision XT8.0 ^6^.

*Bright Open Field test (BOF - AU)*: Mice were placed individually in an open field (60×60×30 cm; 150 lux) for 5 min. Their movements were video-recorded, and the time, spent in the central zone (15×15 cm), was measured by Ethovision XT8.0 ^6^.

*Light and dark box (LDB - AU)*: LDB includes an open white box (30×30×30 cm; 150 lux), interconnected with a dark closed black box (30×30×30 cm). Mice were placed individually in the light box facing the tunnel towards the dark box, and were allowed to move freely for 5 min. Video recordings were scored manually for the amount of time spent in each box, and the number of entries into each box.

*Elevated Plus Maze (EPM - AU)*: EPM has two open arms (30×5cm; 60 lux) and two closed arms (30×5 cm; 15 lux) with 15 cm high non-transparent walls. Mice were placed in the central square (5×5 cm) facing one of the closed arms. Their movements were video-recorded for 5 min., and were scored manually for the amount of time, spent in open arms, and for the number of entries into each arm ^7^.

*Fear Conditioning System (FCS - AU)*: Context-dependent and cue-dependent learning and persistent anxiety of *Brd1*^+/-^ mice were tested using the automated TSE FCS v8.06 (TSE systems GmbH, Bad-Homburg, Germany). FCS includes conditioning, context extinction, cue extinction, and extinction retrieval phases, which span over four days. Principal index of learning was freezing behavior, defined by the absence of any visible movements except those required for respiration ^8^.

*8-Arm Radial Maze (8ARM - AU)*: 8ARM had eight gated arms (30×5 cm) with 15 cm high non-transparent walls radiating from an octagonal central platform, and multiple fixed intra-maze cues. Mice were food deprived for 16 hours/day, until they reached approximately 85% of their free-feeding bodyweights. Our protocol included three training days (15 min/day) and ten trials (one trial/day), during which only three specific arms were baited. Trials were terminated, when mice completed eating all three food pellets or after a delay of 10 min. Video recordings were scored by Ethovision XT8.0 ^6^. Working memory and long-term reference memory errors were defined by the number of re-entries to baited arms, and the number of entries to never-baited arms ^9^.

*24 Hours Locomotion (24HLM - AU)****:*** Mice were placed individually in Makrolon type-III (42.5×26.5×15 cm) cages with free access to food and water at 9am on the test day. Until next day 9am, their movements were monitored by TSE infrared light beam frames and TSE FCS v8.06 (TSE systems GmbH, Bad-Homburg, Germany).

*Prepulse inhibition (HLu)****:*** Prepulse inhibition (PPI) testing was performed using the SM100 Startle Monitor System (Kinder Scientific, Poway, CA, USA), consisting of 8 sound-attenuated startle chambers and StartleMonitor software (Kinder Scientific). Testing procedure was as described elsewhere ^10^. Mice were tested in the setting two times. Day 1 for habituating to the test. Day 3 for testing baseline PPI.

A*mphetamine (AIH) or Cocaine Induced Hyperactivity (CIH) (AU):* Mice were placed individually in Makrolon type-III cages at 9am on the test day. After two hours, one batch of mice were injected with amphetamine (D-Amphetamine, Sigma-Aldrich, St. Louis, USA) 5 mg/kg SC ^11^, and another batch were injected with cocaine either 15 or 30 mg/kg SC. There were corresponding control groups that received only saline injections. Subsequent movements were monitored by TSE infrared frames and TSE FCS v8.06 for four hours.

*High-Performance Liquid Chromatography (HPLC)****:*** Frontal cortical-, hippocampal-, and striatal tissues were collected by free-hand dissection in age-matched male and female mice in parallel. Male data has been reported on elsewhere ^1^. After weighing, samples were homogenized in ice-cold 0.05M HClO_4_ (Sigma-Aldrich), and centrifuged (20000×g) for 30 min. at 4°C. Supernatants were filtered (0.22 µm column; Millipore, Billerica, USA), and were separated by HPLC (ODS 150×2 mm column; flow rate 0.2 ml/minute). Dopamine and serotonin were electrochemically detected (E2=200 mV) by Coulochem-III (Thermo scientific, Sunnyvale, USA).

*Estimation of brain volumes****:*** Female *Brd1*^+/-^ and WT mice (n=7/group) were perfusion fixed through the aorta and brains were removed and post-fixed for at least 48 hours (4% w/v paraformaldehyde in 0.2 M phosphate buffer) and cryoprotected (30% w/v sucrose in PBS) until saturated. Whole brains were sectioned (25 µm coronal sections) and sampled using Systematic Uniform Random Sampling (SURS) with first section randomly selected within the sampling interval. Every 15th section was sampled for Giemsa staining and volume estimation. Total brain volume (hemispheres assessed separately) and ventricular volumes were estimated by systematic point counting using a Nicon eclipse 8oi equipped with an Olympus DP72 camera at a magnification of 1X (hemispheres) and 2X (ventricular system). The counting grid had an area per point of 1.075 mm^2^ (0.018 mm^2^ for the ventricles). Approximately 100–300 points were counted in each region (**Figure S4A**). The total volume was estimated applying the Cavalieri estimator: V(ref)= k∙ t∙ a(p)∙∑P where k is the inverse sampling fraction, t the average section thickness, a(p) the area associated with each point of the grid and ∑ P the total number of points hitting the region of interest. The average section thickness was calculated as the block advance measured by a digital caliper. No efforts were conducted to correct for differential tissue deformation caused by fixation or further tissue processing.

# Supplemental Tables

## Table S1: Basic neurological functioning and behaviors in male and female *Brd1*^+/-^ mice

| **Test** | **Parameters** | ♂ | ♀ | **Implication** |
| --- | --- | --- | --- | --- |
| Irwin's observational battery | Undisturbed behavior | - | - | Basic neurological functioning |
|  | Finger approach | - | - | Basic neurological functioning |
|  | Touch escape | - | - | Basic neurological functioning |
|  | Grip strength | - | ↓ | Basic neurological functioning |
|  | Visual placing response | - | - | Basic neurological functioning |
|  | Corneal response | - | - | Basic neurological functioning |
|  | Toe-pinch response | - | - | Basic neurological functioning |
|  | Wire-maneuver | - | ↓ | Basic neurological functioning |
|  | Limb- and abdominal tone | - | - | Basic neurological functioning |
|  | Tail-pinch response | - | - | Basic neurological functioning |
| Hot-plate | Response | - | - | Acute pain response |
| Hidden food retrieval | Time to retrieve | - | ND | Olfactory functioning |
| Beam walking | Crossing speed/missteps | - | - | Motor coordination |
| Rota-rod | Latency to fall | - | ↓ | Motor coordination |
| Foot-printing test | Stride length | - | - | Motor coordination |
|  | Base width | - | - | Motor coordination |
|  | Step uniformity | - | ↓ | Motor coordination |
| Social interaction | Passive interaction | ↓ | ND | Associability |
|  | Aggressive interaction | ↑ | ND | Aggression |
|  | Latency to interaction | ↑ | ND | Social withdrawal |
| 3 chamber test | Sociability | ↓ | ND | Social withdrawal |
|  | Social recognition | - | ND | Social cognition |
|  | Remote social memory | ↓ | ND | Long term recognition memory |
| Spontaneous alternation (SA) | Baseline | - | ND | Working memory |
|  | PCP induced | ↓ | ND | Working memory |
| Continuous alternation (CA) | Baseline | - | ND | Working memory |
|  | PCP induced | ↓ | ND | Working memory |
| Fear Conditioning (FCS) | Conditioning | ↓ | ↓ | Conditional learning |
|  | Contextual memory (day 2) | ↓ | ↓ | Associative memory’ |
|  | Contextual memory (day 3) | ↓ | ND | Associative memory* |
|  | Contextual memory (day 7) | ↓ | ND | Associative memory* |
|  | Extinction retrieval | - | - | Associative memory |
|  | Cue dependent learning | ↓ | - | Associative memory |
| Acoustic startle reactivity (ASR) | Startle | ↑ | ↑ | Hearing/stress susceptibility |
|  | Latency to startle | ↓ | ↓ | Stress susceptibility |
| Prepulse inhibition (PPI) | Baseline | - | ↓ | Pre-attentive processing |
|  | PCP induced | ↓ | ND | Pre-attentive processing |
|  | Amphetamine induced | - | ND | Pre-attentive processing |
| Locomotor activity | Novelty-induced | - | - | Psycho-motor activity |
|  | PCP induced | ↑ | ND | Cortico-thalamic/Meso-limbic drug responsiveness |
|  | Amphetamine induced | - | - | Meso-limbic drug responsiveness |
|  | Cocaine-induced | ↑ | - | Meso-limbic drug responsiveness |
| Delayed alternation (DA) |  | ↓ | ND | Working memory/spatial reference memory |
| 8 arm radial maze (ARM) | Re-entry to baited arms | ↑ | - | Working memory |
|  | Entry to non-baited arms | ↑ | ↑ | Non-spatial reference memory |
| Morris water maze (MWM) | Acquisition | - | ND | Learning |
|  | Probe test | - | ND | Spatial reference memory |
|  | Flag test | - | ND | Vision |
| Attentional set shifting (ASST) | Rule learning | ↓ | ND | Learning |
|  | Reverse learning | - | ND | Reverse learning |
|  | Intradimensional shift | - | ND | Executive functioning |
|  | Extradimensional shift | ↓ | ND | Executive functioning |
| Elevated plus maze (EPM) | Time in open arms | - | - | Anxiety behavior/Mania |
| Bright open field (BOF) | Time in central zone | - | - | Anxiety behavior/Mania |
| Light and dark box (LDB) | Time in light box | ↓ | - | Anxiety behavior/Mania |
| Open field test (OF) | Distance moved | - | - | Anxiety behavior/Mania |
| Forced swim test (FST) | Immobility | - | ↑ | Behavioral despair/Mania |
| Tail suspension test (TST) | Immobility | - | ↑ | Behavioral despair/Mania |
| Sucrose preference test (SPT) | Sucrose preference | ND | ↓ | Anhedonia |
| PTZ induced seizure activity | Myoclonic jerks (#) | ↑ | ND | Sensitivity of GABA A receptor response (local) |
|  | Clonic seizures (#) | ↑ | ND | Sensitivity of GABA A receptor response (global) |
|  | Clonic seizures (onset) | ↓ | ND | Sensitivity of GABA A receptor response (global) |
|  | Clonic-tonic seizures | - | ND | Sensitivity of GABA A receptor response (global) |

Data presented in this manuscript are highlighted in grey, whereas other data have previously been reported on ^1,12^**. #:** number of events**.** ND: not determined. *Likely reflect acquisition deficit during conditioning.

## Table S2: AMG DEG analysis of female *Brd1*^+/-^ vehicle vs wild type vehicle

See appendix 1

## Table S3: Canonical IPA analysis of AMG DEGs between female *Brd1*^+/-^ vehicle vs wild type vehicle

See appendix 1

## Table S4: Upstream IPA analysis of AMG DEGs between female *Brd1*^+/-^ vehicle vs wild type vehicle

See appendix 1

## Table S5: DEG analysis of AMG DEGs between female *Brd1*^+/-^ FLX vs *Brd1*^+/-^ vehicle

See appendix 1

## Table S6: AMG DEG analysis of female *Brd1*^+/-^ IMN vs *Brd1*^+/-^ vehicle

See appendix 1

## Table S7: aCC DEG analysis of female *Brd1*^+/-^ vehicle vs wild type vehicle

See appendix 1

## Table S8: Canonical IPA analysis of aCC DEGs between female *Brd1*^+/-^ vehicle vs wild type vehicle

See appendix 1

## Table S9: aCC DEG analysis of female *Brd1*^+/-^ IMN vs *Brd1*^+/-^ vehicle

See appendix 1

## Table S10: CPu DEG analysis of female *Brd1*^+/-^ vehicle vs wild type vehicle

See appendix 1

## Table S11: Canonical IPA analysis of CPu DEGs between female *Brd1*^+/-^ vehicle vs wild type vehicle

See appendix 1

## Table S12: Upstream IPA analysis of CPu DEGs between female *Brd1*^+/-^ vehicle vs wild type vehicle

See appendix 1

## Table S13: CPu DEG analysis of female wild type IMN vs wild type vehicle

See appendix 1

## Table S14: CPu DEG analysis of female *Brd1*^+/-^ IMN vs *Brd1*^+/-^ vehicle

See appendix 1

# Supplemental Figures

A B

C D E F

G H

Figure S1.Basic neurological functioning and motor coordination**. A)** Hotplate: *Brd1*^+/-^mice displayed normal reaction time in a pain response test (WT n=14, *Brd1*^+/-^ n=13) **B)** Rotarod: Female *Brd1*^+/-^ mice fell off the rotating rod faster than their WT littermates (2-WAY ANOVA, genotype effect, F_1, 4_ = 15,39, *p*=0.017 ). **C)** Gait in *Brd1*^+/-^ mice was analyzed in their foot print pattern. No difference was observed between *Brd1*^+/-^ and WT mice in stride length, **D)** front base, **E)** hind base. **F)** However, *Brd1*^+/-^ mice displayed slightly disrupted gaiting uniformity as measured by separation between front and hind paw prints (WT n=13, *Brd1*^+/-^ n=12; t=2.19; *p*=0.040). **G)** Beamwalk: no difference was observed between genotypes in the balance beam walking task in regard to missteps, **H)** nor latency to cross the beam. *: p<0.05

**A**


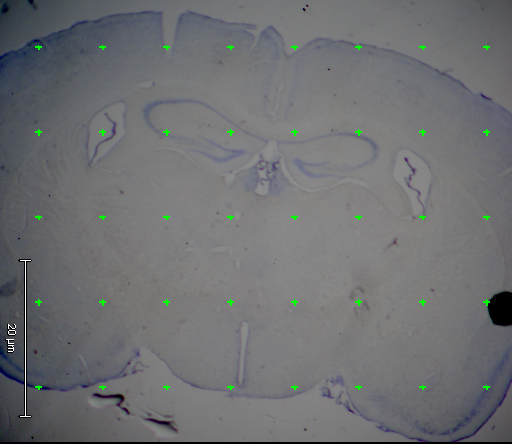

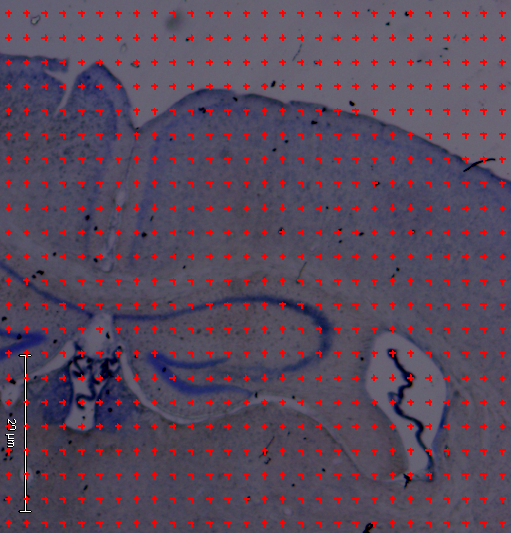


**B C D**

Figure S2. Brain and neuron morphometry in *Brd1*^+/-^ mice. **A)** Example of grid density in brain (L) and ventricle (R) volume estimation. **B)** Symmetry of volumes in left and right hemisphere did not differ between *Brd1*^+/-^ (n=7) and WT (n=7) mice. **C)** Total estimated lateral ventricle volume did not differ between *Brd1*^+/-^ (n=7) and WT (n=7) mice. **D)** Total estimated 3rd ventricle volume did not differ between *Brd1*^+/-^ (n=7) and WT (n=7) mice.

**
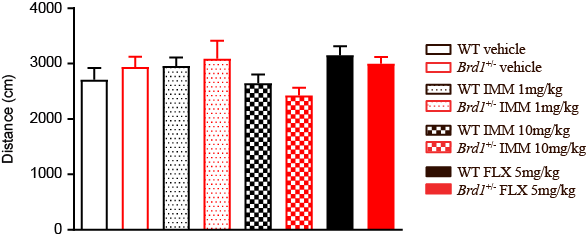
**

*

Figure S3. Open field test on *Brd1*^+/-^ and WT mice following injection with vehicle, IMN or FLX. Vehicle-treated female *Brd1*^+/-^ and WT mice did not differ significantly in their movements in OF. Although 10mg/kg IMN injection significantly reduced activity in *Brd1*^+/-^ mice compared to vehicle injection (t=2.25; p=0.03), no difference in activity was seen between treated *Brd1*^+/-^ and treated WT mice in any of the groups (IMN 1 mg/kg, t=0.36; p=0.72: IMN 10 mg/kg,: FLX 5mg/kg, *: p<0.05

# Supplemental References

1 Qvist P *et al.* The Schizophrenia-Associated BRD1 Gene Regulates Behavior, Neurotransmission, and Expression of Schizophrenia Risk Enriched Gene Sets in Mice. *Biol Psychiatry* 2017; **82**: 62–76.

2 Irwin S. Comprehensive observational assessment: Ia. A systematic, quantitative procedure for assessing the behavioral and physiologic state of the mouse. *Psychopharmacologia* 1968; **13**: 222–57.

3 Bale TL, Vale WW. Increased depression-like behaviors in corticotropin-releasing factor receptor-2-deficient mice: sexually dichotomous responses. *J Neurosci* 2003; **23**: 5295–301.

4 Cryan JF, Mombereau C, Vassout A. The tail suspension test as a model for assessing antidepressant activity: review of pharmacological and genetic studies in mice. *Neurosci Biobehav Rev* 2005; **29**: 571–625.

5 Overstreet DH. Modeling depression in animal models. *Methods Mol Biol* 2012; **829**: 125–44.

6 Noldus LP, Spink AJ, Tegelenbosch RA. EthoVision: a versatile video tracking system for automation of behavioral experiments. *Behav Res Methods Instrum Comput* 2001; **33**: 398–414.

7 Walf A a, Frye C a. The use of the elevated plus maze as an assay of anxiety-related behavior in rodents. *Nat Protoc* 2007; **2**: 322–8.

8 Whittle N, Hauschild M, Lubec G, Holmes A, Singewald N. Rescue of impaired fear extinction and normalization of cortico-amygdala circuit dysfunction in a genetic mouse model by dietary zinc restriction. *J Neurosci* 2010; **30**: 13586–96.

9 Zlomuzica A, Ruocco L, Sadile A, Huston J, Dere E. Histamine H1 receptor knockout mice exhibit impaired spatial memory in the eight-arm radial maze. *Br J Pharmacol* 2009; **157**: 86–91.

10 Fejgin K *et al.* A Mouse Model that Recapitulates Cardinal Features of the 15q13.3 Microdeletion Syndrome Including Schizophrenia- and Epilepsy-Related Alterations. *Biol Psychiatry* 2013; : 1–10.

11 Yates JW, Meij JTA, Sullivan JR, Richtand NM, Yu L. Bimodal effect of amphetamine on motor behaviors in C57BL/6 mice. *Neurosci Lett* 2007; **427**: 66–70.

12 Qvist P *et al.* Mice heterozygous for an inactivated allele of the schizophrenia associated Brd1 gene display selective cognitive deficits with translational relevance to schizophrenia. *Neurobiol Learn Mem* 2017; **141**: 44–52.
